# The novel gene, *mtre-1* , is expressed downstream of MAB-3 and DMD-3 in the male tail tip at the termination of male tail tip retraction.

**DOI:** 10.17912/micropub.biology.000976

**Published:** 2023-10-18

**Authors:** Megan Lesperance, Antonio Herrera, David H.A. Fitch, D. Adam Mason

**Affiliations:** 1 Biology, Siena College, Albany, New York, United States; 2 Department of Biology, Center for Developmental Genetics, New York University, New York, New York, United States

## Abstract

The development of the adult *C. elegans* male tail involves an extensive remodeling during the last larval stage where the pointed tail of the L4 male is converted into the blunt-ended adult tail with its collection of mechano-sensitive rays. The first step in this remodeling is the retraction of the four hypodermal cells of the tail tip to generate the blunt-ended tail. Male tail tip retraction is an excellent model for characterizing how upstream regulatory networks interact with the downstream cell biological effectors that drive morphogenetic changes in all animals. Previously, we’ve shown that two DM-domain transcription factors, MAB-3 and DMD-3 , are central regulators of male tail tip retraction. Using a microarray-based approach we have identified ~400 genes that are more highly expressed in the L4 male tail tip relative to the hermaphrodite L4 tail tip. The uncharacterized gene *T05H10.3* , which we’ve named *mtre-1* , was highly over-represented in the male tail tip vs. the hermaphrodite tail tip and was under-represented in *mab-3 ; dmd-3* mutant male tail tips vs. wild-type male tail tips. A transcriptional reporter for *mtre-1* shows clear expression in the male tail tip cells for a short period (~3 hours) at the end of retraction. This expression is dependent on the activity of MAB-3 and DMD-3 , since expression is reduced in *dmd-3* single mutant males and absent in *mab-3 ; dmd-3* mutant males. Finally, males homozygous for a putative null allele of *mtre-1* display a phenotypically wild-type adult male tail, indicating that *mtre-1* is not essential for male tail morphogenesis.

**Figure 1. mtre-1 is expressed in the male tail tip at the end of retraction:**
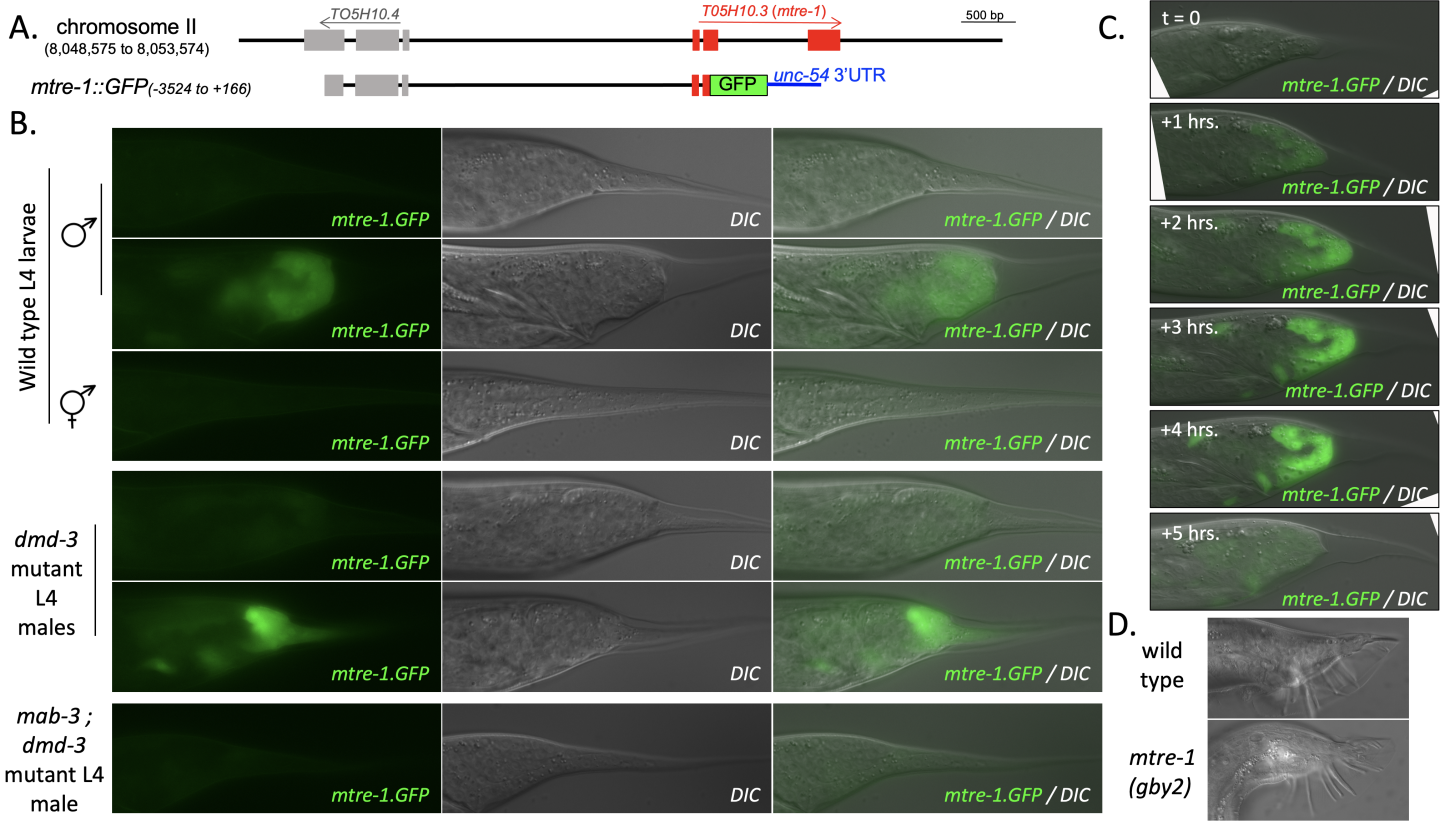
(A) Diagram of chromosome II in the *mtre-1* region and the *mtre-1 ::GFP* reporter transgene. Exons are indicated with boxes and nucleotide numbers in the reporter are relative to the A TG start codon of *mtre-1* . (B) GFP, DIC and GFP/DIC overlay images of *mtre-1 :* :GFP in wild type, *dmd-3* mutants and *mab-3 ; dmd-3* mutant mid-L4 larvae. A younger L4 wild type male larva that is beginning retraction and an older L4 wild type male larva that is completing retraction are shown to illustrate that *mtre-1 ::GFP* expression does not initiate until the very end of retraction in wild type males. Two *dmd-3* mutant larvae of approximately the same age are shown to illustrate the observed incomplete penetrance of expression level change. (C) GFP/DIC overlay images of *mtre-1 ::GFP* expression in a single wild-type L4 male over the course of 5 hours demonstrating the dynamics of *mtre-1 ::GFP* expression in the male tail tip. (D) DIC images of adult tails of a wild-type male and a male homozygous for the *gby2* putative null allele of *mtre-1* showing that *mtre-1* mutant tails are wild-type in appearance.

## Description

The development of a single-celled fertilized egg into a multicellular adult is an essential part of all animal life cycles. Morphogenesis, a critical component of development, is the process by which embryonic structures are sculpted to generate the final adult form. Morphogenesis is driven by changes in the migration and shape of embryonic cells and tissues. While much is known about the cellular processes that function to alter the adhesion, shape and motility of cells during morphogenesis, much less is known about how these processes are regulated to ensure that they occur in the correct time and place.

The remodeling of the tail of *C. elegans* males during the last larval stage serves as an excellent model to study the regulatory and cell biological processes that underlie morphogenesis. During the last larval stage, males undergo a number of sex-specific developmental events to produce the sexually dimorphic adult male body form (Nguyen et al., 1999; Emmons, 2005) . The tails of adult male *C. elegans* are blunt-ended and contain extensive sensory structures used during mating. In contrast, the tails of male L1-L4 larvae are pointed. During the L4 larval stage a male-specific remodeling event transforms the pointed larval tail into the adult male tail. A major step in this remodeling is tail tip retraction, during which the four tail tip hypodermal cells (hyp8-11) fuse together, become rounded and retract away from the larval cuticle. The hyp8-11 syncytial cell subsequently migrates anteriorly and internally. Following tail tip retraction, a second retraction event, called anterior tail retraction, produces the final overall shape of the adult male tail, including the fan and rays. In hermaphrodites these morphogenetic events do not occur and the pointed shape of the L4 tail is maintained into adulthood (Nguyen et al., 1999; Emmons, 2005) .

The *dmd-3* gene encodes a DM domain-containing transcription factor that is both necessary and sufficient for tail tip retraction (Mason et al., 2008) . *dmd-3* is expressed male-specifically in hyp8-11 immediately preceding the onset of tail tip retraction and expression persists until retraction is completed. Males that are homozygous for a putative null allele of *dmd-3* display only partial tail tip and anterior tail retraction, resulting in a pointed tail being maintained into adulthood. A second DM domain transcription factor, MAB-3 , acts partially redundantly with DMD-3 in retraction, since *mab-3 ; dmd-3* double-mutant males fail to undergo either tail tip or anterior tail retraction and the overall shape of the L4 tail tip is maintained into adulthood (Mason et al., 2008) . Importantly, DMD-3 appears to occupy a central node in the genetic regulatory network controlling retraction. Current evidence suggests a “bow-tie” regulatory model in which multiple signaling pathways converge upon the regulatory region of *dmd-3* to drive expression at the correct time, sex and location (Nelson et al., 2010) . DMD-3 then presumably regulates the transcription of multiple cell biological effector genes that carry out morphogenesis.

Male tail tip retraction provides a unique system for identifying novel morphogenetic genes and regulatory mechanisms because: i) *C. elegans* is remarkably easy to manipulate genetically, ii) this process is well characterized morphologically (Nguyen et al., 1999) , iii) male tail tip retraction involves changes in only four cells, making it a very simple model for morphogenesis, iv) Since DMD-3 is a master regulator of male tail tip retraction, characterizing the genes that lie downstream of DMD-3 will allow high-resolution characterization of mechanisms that regulate effectors of morphogenesis. Here we report the results of a microarray based approach that identified ~400 putative MAB-3 / DMD-3 regulated genes. The *mtre-1* gene was identified as one of the top genes displaying male-biased expression in wild-type tail tips. An *mtre-1* ::GFP transcriptional reporter is expressed downstream of MAB-3 / DMD-3 activity in the male tail tip hypodermal cells for a brief period of time at the very end of retraction. Finally, a putative null allele of *mtre-1* was generated. Homozygous mutant males display a wild-type appearing tail tip, suggesting that *mtre-1* is not required for tail tip retraction.

To identify genes that show sexually dimorphic expression in tail tip dependent on *mab-3* and/or *dmd-3* activity we performed a microarray screen using mRNAs purified from tail tips laser dissected from wild-type (normal) males, *mab-3 ; dmd-3* mutant males and wild-type hermaphrodites. This process identified 392 genes that showed significant differences in expression levels in male and hermaphrodite tail tips, 71 of which also displayed significant differences between wild-type males and *mab-3 ; dmd-3* mutant males. The gene *T05H10.3* was identified as being the second most male-biased gene in the microarray (3.36 times higher in wild-type L4 males relative to wild-type L4 hermaphrodites). Furthermore, *T05H10.3* mRNA levels were greatly reduced in *mab-3 ; dmd-3* mutants with wild-type males showing 2.98 times higher expression relative to the double mutants. *T05H10.3* is an uncharacterized gene with no predicted protein domains or sequence homology to any genes outside of the nematode clade. Orthologs are highly conserved within Rhabditina nematode genomes with relatively high degree of sequence identity (75-99%). Since *T05H10.3* lacks any clear functional domains and is expressed in the retracting male tail tip, we named this gene *mtre-1* for **m** ale **t** ail **r** etraction **e** xpression.

We generated transcriptional reporters for a subset of the 71 putative DMD-3 - and/or MAB-3 -regulated genes and examined their expression patterns. Interestingly, transcriptional GFP reporter transgenes for *mtre-1* are preferentially expressed in L4 male tail tips at the end of tail tip retraction ( Figure 1A, B ). Specifically, 75% (27/36) of wild-type L4 males displayed high levels of *mtre-1 ::GFP* expression at the end of tail tip retraction, 19% (7/36) displayed low levels of expression and 6% (2/36) failed to show expression. In contrast, 81% (30/37) of wild-type L4 hermaphrodites failed to show any *mtre-1 ::GFP* expression in the tail tip, with the remaining 19% (7/37) only showing faint expression. To more closely examine the dynamics of *mtre-1 ::GFP* expression, we followed single wild-type L4 males throughout the process of retraction. We observe that *mtre-1 ::GFP* expression in the male tail tip is restricted to a short 3- to 4-hour window that initiates after tail tip retraction is almost completed and terminates at the onset of anterior tail retraction. In contrast, we did not observe high levels of expression in wild-type L4 hermaphrodites at any time point. In addition, we did not observe any other major regions of expression of *mtre-1 ::GFP* in either L4 males or hermaphrodites.

We next asked whether *mtre-1* expression is downstream of DMD-3 and MAB-3 activity by crossing *mtre-1 ::GFP* reporters into single and double mutant backgrounds. Expression of *mtre-1* is clearly reduced in *dmd-3* mutant males as only 35% (7/20) of *dmd-3* mutant L4 males showed high levels of expression in the tail tip, 50% (10/20) showed faint expression and 15% (3/20) showed no expression. The residual *mtre-1 ::GFP* expression observed in *dmd-3* mutants was most likely due to the activity of MAB-3 since 92% (23/25) of *mab-3 ; dmd-3* double mutants failed to express *mtre-1 ::GFP* . We followed single *mab-3 ; dmd-3* mutant males throughout their L4 stage to ensure that we were not missing the window of expression because *mab-3 ; dmd-3* mutants do not undergo retraction and the exact stage can be difficult to determine. Similar to wild-type L4 hermaphrodites, *mab-3 ; dmd-3* mutants failed to show *mtre-1 ::GFP* expression at any point during the L4 stage.

The *mtre-1 ::GFP* expression pattern is unique for genes that lie downstream of MAB-3 and DMD-3 activity since the majority of MAB-3 and/or DMD-3 regulated genes more closely match the expression dynamics of *dmd-3* and *mab-3* , where expression is initiated before or during the onset of retraction and persists throughout the retraction process (Mason et al., 2008; Nelson et al., 2010) . This unique expression pattern of *mtre-1 ::GFP* raised the possibility that *mtre-1* is activated during the late stages of retraction in order to terminate tail tip retraction. If this is true, we would expect loss-of-function *mtre-1* mutants to display an over-retraction phenotype that is commonly observed when retraction does not terminate properly (Del Rio Albrechtsen et al., 2006; Mason *et al* ., 2008; Nelson *et al* ., 2010). To generate a putative null allele of *mtre-1* , we used a CRISPR/CAS genome editing approach to generate an in-frame stop codon at codon 30 in the second exon. Males homozygous for this allele displayed a phenotypically wild-type tail, indicating that *mtre-1* is not required for the termination of male tail tip retraction ( Figure 1D ).

*mtre-1* is part of a nematode specific gene family, and there are 4 additional paralogs in the *C. elegans* genome ( *Y75B12B.1 , C25H3.15* , *C35A5.11* and *C06E7.88* ). Interestingly, a recent RNA sequencing analysis of wild-type and *dmd-3* mutant tail tips identified both *C35A5.11* and *C06E7.88* as being expressed in the male tail tip downstream of DMD-3 activity (manuscript in preparation). This raises the possibility that *mtre-1* , *C35A5.11* and *C06E7.88* function redundantly during male tail tip retraction. An analysis of double and triple mutants will be required to determine if this is the case. In summary, this work has identified a novel uncharacterized gene with a unique and dynamic expression pattern downstream of MAB-3 and/or DMD-3 activity in the male tail tip. While loss-of-function mutants appear to undergo male tail tip retraction normally, it is possible that *mtre-1* functions redundantly with additional paralogs that are expressed in the male tail tip.

## Methods

*Microarray Analysis*

Laser-capture microdissection (LCM) was used to isolate tail tips from worms that were synchronized by hatch-off, as described previously (Woronik et al., 2022) , except bulk quantities of tail tips were collected instead of single tail tips (60-100 tail tips per sample) and worms were collected after a 2-hour instead of 1-hour hatch-off. Worms were fixed in cold 70% methanol and pipetted onto Leica PEN membrane slides and dried prior to dissection. Laser-dissected tail tips were collected in lysis buffer provided with the RNAqueous Micro Kit. Aliquots of 1 µl were run on RNA Pico Chips to test for purity, molecular weight range and sample concentration. RNA was amplified using WT-Ovation Pico RNA Amplification System (Nugen) with the kit protocol. Samples were then biotinylated using the FL-Ovation cDNA Biotin Module. The resulting probe cDNA (5 µg) was hybridized to Affymetrix *C. elegans* GeneChips overnight. Chips were then washed in the Affymetrix fluidics station (at the New York University Genomics Core) and signal was recorded. Two biological replicates were used for each type of sample (mid-L4 wild-type males, wild-type hermaphrodites, and *mab-3 ; dmd-3* males). Two comparisons were used to find genes that were sex-specific (males vs. hermaphrodites) and downstream of *dmd-3* and/or *mab-3* (WT vs. *dmd-3 ; mab-3* males). SAM (Statistical Analysis of Microarrays; Tusher et al., 2001) was used to analyze microarray data to identify differentially-expressed genes. After normalizing the expression values, we used a *t* -test with a >2-fold change threshold to identify significantly differentially-expressed genes. Our tail tip-specific analysis yielded 392 sex-specific and 172 *dmd-3 ; mab-3* -dependent genes, with an intersection of 71 genes.

*mtre-1* ::GFP *reporter transgene*

To generate a transcriptional reporter of *mtre-1* , the upstream regulatory region between -3,524 nucleotides upstream to +166 nucleotides downstream of the **A** TG start codon was PCR amplified with the addition of a 5’ *BamHI* site and a 3’ *KpnI* site ( Figure 1A ). The PCR product was digested with *BamHI/KpnI* and ligated into the pPD95.75 plasmid to generate pCNA34 [ *T05H10.3* (-3524 to +166)::GFP:: *unc-54* 3’UTR] plasmid. This plasmid was injected into *pha-1 ( e2123 ) III ; him-5 ( e1490 )V* to generate the *gbyEx37* [ pCNA34 ( *mtre-1 ::GFP* ); *pBXI ( pha-1 (+)]; pha-1 ( e2123 ) III ; him-5 ( e1490 )V* transgenic strain.

*mtre-1 ( gby2 ) putative null allele.*

To generate a putative null allele of *mtre-1* we utilized the general approach described in Ward, 2015. Briefly, the guide sequence of ccaacaaactctatccgtgg present in exon 1 of *mtre-1* was chosen using the MIT guide selector website [http://crispr.mit.edu/]. The guide sequence and the tra-crRNA were placed downstream of the U6 promoter using overlapping PCR (Ward, 2015) . A 80 nucleotide repair template was designed that changed the sequence: CGT GGA GGA CCA to C *TC* ***TAG*** *A* GA C CC A. These changes generated an in-frame stop codon (bold), an XbaI site for detection of the succesful edit (italics), and an extra nucleotide insertion to generate a frameshift mutation (underline). We utilized a *pha-1* co-conversion method to generate our mutant allele. Briefly, the *mtre-1* guide containing PCR + the *mtre-1* repair template, + the pJW1285 plasmid + a *pha-1* + repair template was injected in the *pha-1 ( e2123 )* temperature senstive adult hermaphrdoites and *pha-1 +* edited F1s were selected for survival at 25 ^o^ C. *mtre-1* PCR products amplified from converted strains were digested with *XbaI* to identify strains that contained the edited allele of *mtre-1* .

*Microscopy*

DIC and fluorescent images were generated using a Nikon Ti-S compound inverted microscope equipped with a Hamamatsu C11440-10C Flash 2.8 camera. The NIS Elements Version 4.2 software was used for image acquisition. All digital images were processed using the Adobe Photoshop Elements Version 24.5.0 software (Adobe Systems, San Jose, CA). Animals utilized for examination by microscopy were either picked from mixed staged plates or from bleach synchronized populations. Worms were bleach synchronized as previously described with minor modification (Lewis and Fleming, 1995) .

## Reagents

**Reagents**

*C. elegans* strains

**Table:** 

Strain	genotype	description
*GE24*	*pha-1 ( e2123 )III *	*pha-1* temperature sensitive strain. Used for generating *mtre-1* null allele.
*EM574*	*pha-1 ( e2123 )III; him-5 ( e1490 ) V *	*pha-1* temperature sensitive strain with *him-5* mutant allele. Used for generating transgenic strains.
*CNA53*	*gbyEx37 [ pCNA34 ( mtre-1 ::GFP) ; pBXI ( pha-1 (+)] ; pha-1 ( e2123 )III; him-5 ( e1490 ) V *	*mtre-1 ::GFP* transcriptional reporter in a wild-type background
*CNA132*	*gbyEx37 [ pCNA34 ( mtre-1 ::GFP) ; pBXI ( pha-1 (+)] ; pha-1 ( e2123 )III; him-5 ( e1490 ), dmd-3 ( ok1327 )V *	*mtre-1 ::GFP* transcriptional reporter in a *dmd-3* single mutant background
*CNA130*	*gbyEx37 [ pCNA34 ( mtre-1 ::GFP) ; pBXI ( pha-1 (+)] ; mab-3 ( e1240 ) II ; pha-1 ( e2123 )III; him-5 ( e1490 ), dmd-3 ( ok1327 )V *	*mtre-1 ::GFP* transcriptional reporter in a *mab-3 / dmd-3* double mutant background
*CNA232*	*mtre-1 ( gby2 ) II; qIs56 [ lag-2 ::GFP], him-5 ( e1490 ) V *	*mtre-1* putative null allele crossed to *him-5 ( e1490 ) *

Plasmids / PCR products

**Table:** 

**Plasmid / PCR products**	**genotype**	**description**
*pCNA34*	*mtre-1 promoter (-3524 to +166)::GFP:: unc-54 3'UTR *	Plasmid containing the *mtre-1* promoter region ligated into *BamHI / KpnI* sites in *pPD95.75*
*MLPCR34*	* U6 promoter:: mtre-1 guide RNA::tracrRNA *	Overlap PCR product containing the *mtre-1* guide RNA template fused to tracrRNA template downstream of U6 promoter

Transgenes

**Table:** 

**transgene**	**genotype**	**description**
*gbyEx37*	*pCNA34 ( mtre-1 ::GFP) ; pBXI ( pha-1 (+)] *	Contains *mtre-1 ::GFP* reporter + *pha-1 +* for selection of transgenic strains
